# Urinary Schistosomiasis with Simultaneous Bladder Squamous Cell Carcinoma and Transitional Cell Carcinoma

**Published:** 2012

**Authors:** AA Ketabchi, GR Moshtaghi-Kashanian

**Affiliations:** 1 Department of Urology, Physiology Research Center, Kerman Medical Sciences University, Kerman, Iran; 2 Department of Biochemistry, Medical School, Kerman University of Medical Sciences, Kerman, Iran

**Keywords:** *Schistosoma haematobium*, Bladder, Squamous cell carcinoma, Transitional Cell

## Abstract

This is a case report concerning a 60 years old man who lived for a short period in an endemic area of Khuzestan Province (neighboring province of Persian Gulf in Iran) for approximately 20 years ago. Recently he referred to the Urology Department of Kerman University of Medical Sciences with hematuria and dysuria. In the sonography a polypoid mass on the bladder floor was observed. In the cystoscopy and biopsy a bladder tumor (Simultaneous squamous cell carcinoma and Transitional Bladder Cell Carcinoma) and schistosomiasis (*Schistosoma haematobium*) was diagnosed.

## Introduction

Schistosomiasis is a parasitic and endemic disease in more than 75 countries while more than 200 million of world populations are infested to this parasite (1, 2) This parasitic chronic disease caused by a blood circulation parasite known as *Schistosoma haematobium*. This infection induces chronic inflammation in the urinary tract, most likely bladder and rarely in female genital in endemic area due to the deposition of large numbers of eggs in the sub-epithelial tissues (3). The urinary tract infested patients are suffering chronic granulomatosis with the clinical symptoms of urinary frequency, dysuria and terminal hematuria. If urinary schistosomiasis infested patients left untreated it may cause kidney failure, bladder cancer, and even prostate cancer (4, 5).

One of the main consequences of infection with *S. haematobium* is a marked increase in the incidence of carcinoma of the bladder, although this bladder cancer link in schistosomiasis is generally accepted but its carcinogenic mechanisms are less clear (6).

The predominant type of bladder cancer in schistosomiasis is squamous cell carcinoma and also the age range of schistosomiasis dependent bladder cancers patients are lower than non schistosomiasis bladder cancers (third or fourth decade vs. seventh) (2).

So far there were rare reports regarding simultaneous incidence of squamous cell and transitional bladder cell carcinoma.

## Case presentation

**Fig. 1:**
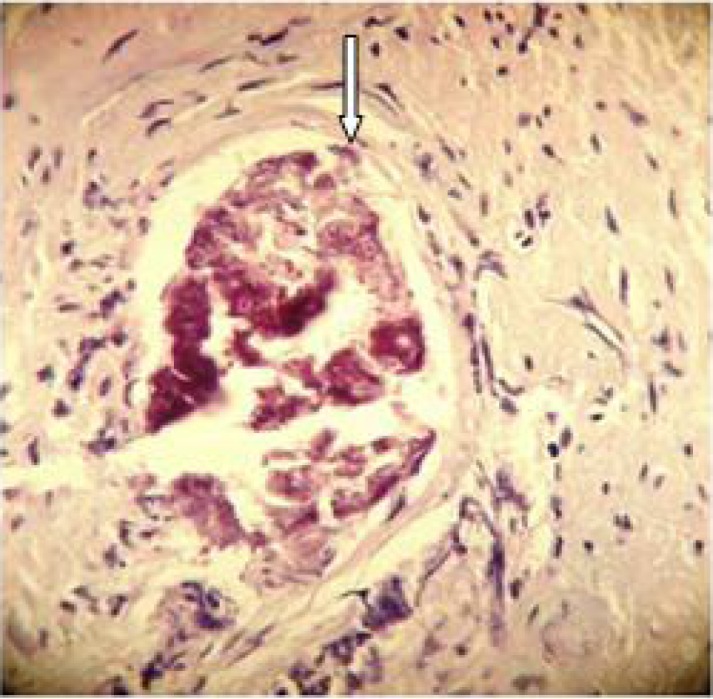
Calcified *Schistosoma* ova in fibro muscular stroma with characteristic terminal spine (Arrow)

**Fig. 2:**
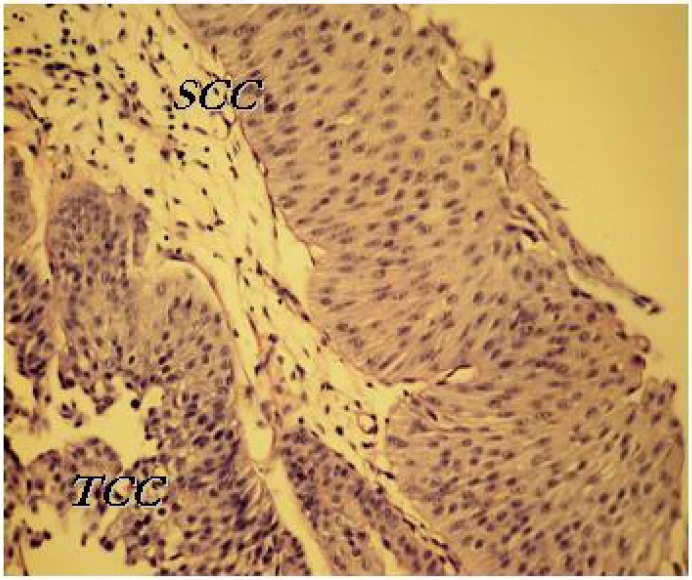
Squamous cell carcinoma (superficial), transitional cell carcinoma (in deeper lay)

**Fig. 3:**
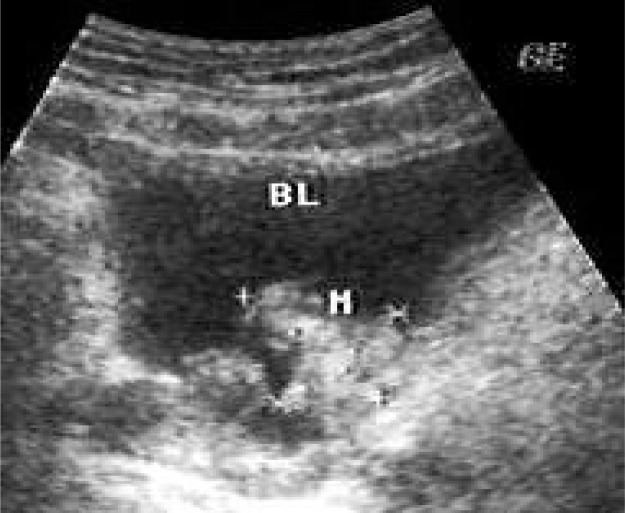
Polypoid mass in bottom of bladder (M)

This patient was a 60-years-old man who attended urology outpatient clinic of our teaching hospital in May 2008 with a 3-month history of features of urinary frequency, dysuria and hematuria. There was no history of diabetes, sexually transmitted infection or trauma. Also he had a short stay history about 20 years ago in Khuzestan Province (neighboring province of Persian Gulf in Iran). Khuzestan is the only area in south-west Iran where urinary schistosomiasis was prevalent (7). On examination, his vital signs were essentially normal direct rectal examination showed a mild enlarged prostate with smooth surface and his serum prostatic specific antigen (PSA) was 2.2 ng/mL, which is normal for age/assumed prostatic volume. His preliminary hematological and renal function tests were normal, in suprapubic sonography that was done from his bladder, it showed a polypoid tumor on the floor of his bladder (Fig. 1), after cystoscopy and obtaining three specimens from bladder mass the histopathology study showed simultaneous squamous cell, transitional cell carcinoma (superficial type), and *S. haematobium* (Fig. 2 and 3), and then patient treated classically for bladder cancer (TUR-T, locally chemotherapy) and schistosomiasis (Praziquantel). After nearly 7 months treatment and follow-up (cystoscopy and biopsy), there was not any sign of disease.

## Conclusion

Bladder tumors related to schistosomiasis are mainly squamous cell type; there are rare reports of simultaneous presence of both tumors. It was also interesting to observe schistosomiasis with such tumors in the bladder of an old patient in a non endemic area. So in presence of simultaneous bladder squamous cell carcinoma and transitional cell carcinoma the *Schistosoma* contamination should be considered too.
